# Defensive functions of volatile organic compounds and essential oils from northern white-cedar in China

**DOI:** 10.1186/s12870-020-02716-6

**Published:** 2020-11-03

**Authors:** Liping Bai, Wenjia Wang, Juan Hua, Zhifu Guo, Shihong Luo

**Affiliations:** 1 College of Bioscience and Biotechnology, Shenyang Agricultural University, Shenyang, 110866 Liaoning Province China; 2 Key Laboratory of Biological Invasions and Global Changes, Shenyang, 110866 Liaoning Province China

**Keywords:** *Thuja occidentalis*, Volatile organic compounds (VOCs), Essential oils (EOs), Phytotoxic effects, Cultivated plant

## Abstract

**Background:**

Plants are known to emit diverse volatile organic compounds (VOCs), which may function as signaling substances in plant communication with other organisms. *Thuja occidentalis*, which is widely cultivated throughout China, releases aromatic VOCs into the air in winter and early spring. The relationship of this cultivated plant with its neighboring plants is necessary for the conservation of biodiversity.

**Results:**

(−)-α-thujone (60.34 ± 5.58%) was found to be the major component in VOCs from the Shenyang population. The essential oils (EOs) from the Kunming and Shenyang populations included the major components (−)-α-thujone, fenchone, (+)-β-thujone, and (+)-hibaene, identified using GC-MS analyses. (−)-α-thujone and (+)-hibaene were purified and identified by NMR identification. EOs and (−)-α-thujone exhibited valuable phytotoxic activities against seed germination and seedling growth of the plants *Taraxacum mongolicum* and *Arabidopsis thaliana.* Moreover, the EOs displayed potent inhibitory activity against pathogenic fungi of maize, including *Fusarium graminearum*, *Curvularia lunata*, and *Bipolaris maydis*, as well as one human fungal pathogen, *Candida albicans.* Quantitative analyses revealed high concentrations of (−)-α-thujone in the leaves of *T. occidentalis* individuals from both the Shenyang and Kunming populations. However, (−)-α-thujone (0.18 ± 0.17 μg/g) was only detected in the rhizosphere soil to a distance of 0.5 m from the plant.

**Conclusions:**

Taken together, our results suggest that the phytotoxic effects and antifungal activities of the EOs and (−)-α-thujone in *T. occidentalis* certainly increased the adaptability of this plant to the environment. Nevertheless, low concentrations of released (−)-α-thujone indicated that reasonable distance of *T. occidentalis* with other plant species will impair the effects of allelochemical of *T. occidentalis*.

## Background

Plant volatile organic compounds (VOCs) that are triggered by both biotic and abiotic stresses can act as a complex mixture of low-molecular weight lipophilic metabolites [1, 2]. Many plant VOCs are the main components of essential oils (EOs) and have had commercial applications, including flavorings and fragrances, since antiquity [3]. Certain VOCs from aromatic plants has been used medicinally to treat conditions that can benefit from their antimicrobial, anti-inflammatory, expectorant, anticonvulsant, analgesic, and spasmolytic activities [1]. Moreover, a primary function of these substances in VOCs and EOs has been demonstrated to be defense against herbivores and pathogens [4–6]. In some plants, emitted VOCs may also function as wound sealers [7]. Additionly, certain released VOCs are also major precursors of phytotoxic compounds which can also act in plant-plant communications [8]. Although it is difficult to study the chemical functions of VOCs and EOs due to pronounced lipophilicity and volatility, the compounds and biological properties of plant VOCs and EOs have been still intriguing research topics.

Of the plant VOCs, the most intensively studied substance group has been the terpenoids [9]. Five-carbon isoprene units, including a wide variety of monoterpenoids and sesquiterpenoids, are emitted from a wide range of species [10]. Terpenoids are predominantly released from plants in sunlight, and broad-leaved trees exhibit strong seasonality in terpenoid emission [11]. The main functions of volatile terpenoids are thought to be efficient protection against a broad range of herbivores, while limiting the chances that the herbivores evolve resistance [12, 13]. A series of sesquiterpenoids were released after herbivore wounding of the fern *Pteris vittata* [14]. Similarly, following damaged by the pest *Diabrotica virgifera virgifera* to the maize roots, maize plants are able to emit (*E*)-β-caryophyllene, which attracts entomopathogenic nematodes [15]. Furthermore, many kinds of terpenoids found in VOCs or EOs have shown phytotoxic activity against seedling root and shoot growth of other plants [16]. The volatile monoterpenoids 1,4-cineole and 1,8-cineole, which have been identified as components in essential oils derived from several plant species, showed significant inhibitory effects on the germination and growth of roots and shoots of *Echinochloa crusgalli* and *Cassia obtusifolia* [17]. Cultivated plants face many of the same challenges as wild plants do, but the plants are often grown outside the native habitat, and cultivation involves selection by humans. A wild plant can be cultivated either to provide sufficient food to meet immediate requirements or to give a satisfactory economic return to humans [18]. However, many introduced plants can cause damage to native biodiversity in their new environments. For example, *Eichhornia crassipes* (Mart.) Solms, native to South America has been cultivated in China as feed for livestock, but has now become major threat to the biodiversity and sustainability of aquatic ecosystems in Dianchi Lake [19]. Similarly, *Ageratina adenophora* (Spreng.) R.M. King & H. Rob, native to Mexico and Central America, initially introduced to China as an ornamental plant, but has now become an invasive weed, seriously damaging the native ecosystems of southwestern China [20, 21]. Thus, the conservation of biodiversity in these cases requires that we study the relationships between exotic plants and native ecosystems.

*Thuja occidentalis* L. (Cupressaceae), commonly known as northern white-cedar, is indigenous to North America [22]. Because of its fragrance, *T. occidentalis* was introduced to Europe as an ornamental tree for a long time as early as the 1540s [23, 24]. More recently, *T. occidentalis* was introduced to many Chinese cities, including Shenyang and Kunming, as an ornamental tree. The trees are propagated by cuttings, and many were grown in this way in the 2000s. Thousands of these cultivated plants planted in Shenyang are now nearly 10 m tall, and have with spreading crowded branches with characteristic vegetation. However, the ground underneath *T. occidentalis* is frequently bare soil without any grass, while vegetation close to these trees is sporadically growing mosses and herbs with obviously inhibited growth. When the leaves of *T. occidentalis* are touched or damaged, a strong aromatic odor with VOCs is released, and is also naturally released by wind in winter and early spring. Because of this, it was thought that volatile growth inhibitors should be released. Thujone was found to be the most abundant constituent of EOs extracted from the leaves of *T. occidentalis* cultivated in Poland [25]. Interestingly, volatile compounds released from *T. occidentalis* exhibited phytotoxic activity against the seeds germination of *Amaranthus caudatus* and *Lepidium sativum* [26]. However, the nature of the involvement of phytotoxic compounds in rhizosphere soil remains unknown, and the allelochemicals in EOs on fungal and plant species needs further research. The aims of this study included to analyze the VOCs and EOs from *T. occidentalis* using GC-MS, and to investigate the biological activities of the EOs and major VOC components.

## Results

### Chemical constituents in VOCs and EOs analyzed using GC-MS

**Fig. 1:**
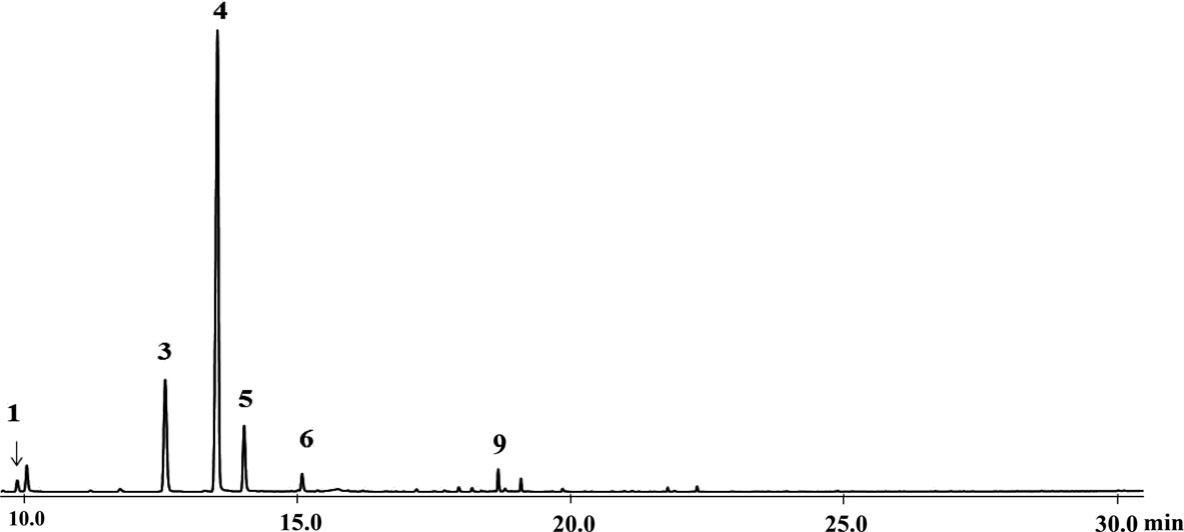
VOCs of *T. occidentalis* from Shenyang population analyzed using GC-MS. ρ-Cymene (**1**), Fenchone (**3**), (−)-α-Thujone (**4**), (+)-β-Thujone (**5**), Camphor (**6**), Fenchyl acetate (**9**)

In order to analyze the secondary metabolites present in *T. occidentalis* VOCs, VOCs were obtained through a closed-loop stripping system. Subsequently, the collected VOCs were directly analyzed by GC-MS. VOCs of Shenyang cultivated population of *T. occidentalis* contained six monoterpenoids, of which (−)-α-thujone was the most abundant monoterpenoid (60.34 ± 5.58%), followed by (+)-β-thujone (23.21 ± 19.62%), and then fenchone (14.00 ± 3.46%) (Fig. 1). Since the amount of VOCs was insufficient for subsequent experiments, EOs from *T. occidentalis* from both Shenyang and Kunming were then collected using the hydrodistillation method [27]. The yield of EOs from the samples taken from the Shenyang and Kunming populations was 0.61 ± 0.03% and 0.41 ± 0.06%, respectively (Table 1). Fourteen terpenoids were identified by GC-MS analyses of EOs from the two investigated populations, and the EOs from the two populations exhibited similar constituents. The dominant compound was found to be (−)-α-thujone, representing 57.44 ± 0.43% and 69.22 ± 10.49% of the total volume of the EOs from Shenyang and Kunming, respectively. Other major components included (+)-β-thujone, fenchone, rimuene, and (+)-hibaene. Diterpenoids are not very volatile, and therefore we did not detect rimuene or (+)-hibaene in the VOCs. To further confirm the chemical structures of constituents, the EOs of *T. occidentalis* in Shenyang were subjected to column chromatography. Interestingly, the two main constituents, (−)-α-thujone [28] and (+)-hibaene [29], were successfully identified after purification by comparison of NMR data with those reported in the literature [30].

**Table 1:** Chemical constitutes of VOCs and EOs of *T. occidentalis*

No	Components	Retention index	% Component in EO and VOC			Identification^a^
			EOs from Shenyang Population	EOs from Kunming Population	VOCs from Shenyang Population	
1	ρ-Cymene	1019	0.55 ± 0.01	0.07 ± 0.10	0.75 ± 0.20	MS
2	γ-Terpinene	1050	0.60 ± 0.02	0.07 ± 0.09	–	MS
3	Fenchone	1082	10.98 ± 0.13	6.35 ± 2.42	14.00 ± 3.46	MS
4	(−)-α-Thujone	1105	57.44 ± 0.43	69.22 ± 10.49	60.34 ± 5.58	S, MS
5	(+)-β-Thujone	1119	6.74 ± 0.04	3.46 ± 0.61	23.21 ± 19.62	MS
6	Camphor	1150	2.21 ± 0.02	1.56 ± 0.13	1.59 ± 0.43	MS
7	(−)-Terpinen-4-ol	1183	3.37 ± 0.01	4.65 ± 0.81	–	MS
8	Pulegone	1199	0.63 ± 0.02	0.84 ± 0.01	–	MS
9	Fenchyl acetate	1286	3.09 ± 0.04	0.28 ± 0.18	0.11 ± 012	MS
10	Bornyl acetate	1292	0.74 ± 0.01	0.85 ± 0.26	–	MS
11	α-Terpinyl acetate	1349	1.28 ± 0.01	0.60 ± 0.33	–	MS
12	Oplopanone	1749	0.51 ± 0.20	0.74 ± 0.35	–	MS
13	Rimuene	1922	4.59 ± 0.23	3.84 ± 4.14	–	MS
14	(+)-Hibaene	1966	7.28 ± 0.35	7.49 ± 7.79	–	S, MS

^a^MS Mass spectra matched with NIST (2014) data

S, the isolated standard

### Seed germination bioassay with EOs and (−)-α-thujone

**Fig. 2:**
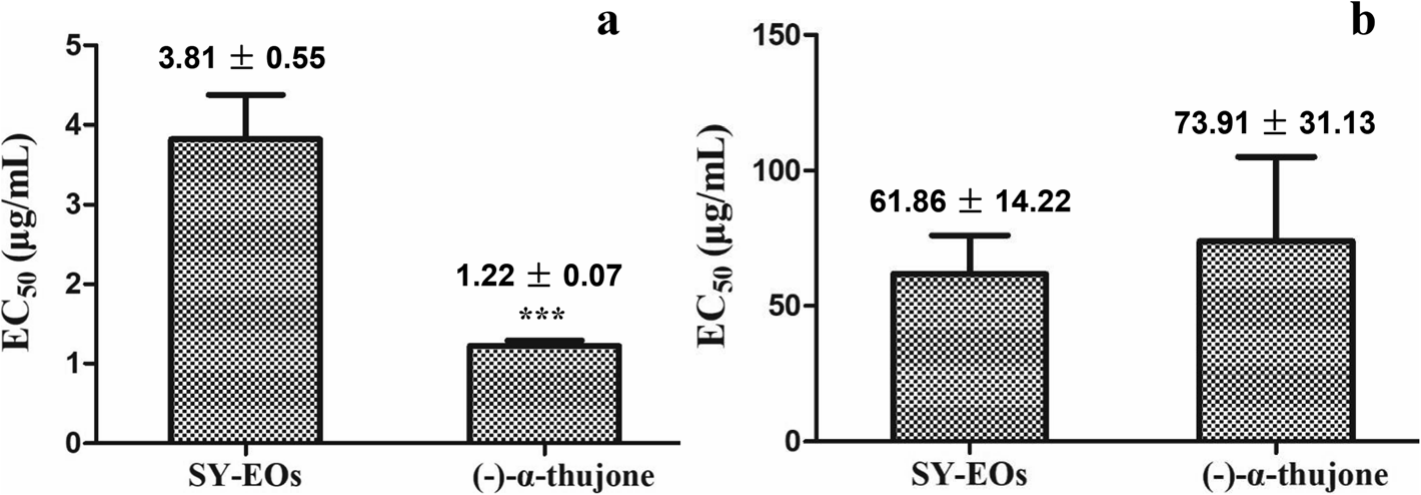
Activity of *T. occidentalis* EOs and (−)-α-thujone against germination of *Arabidopsis thaliana* and *Taraxacum mongolicum* seeds (*n* = 4)*.* **a** Seed germination of *A. thaliana*, (**b**) Seed germination of *T. mongolicum* (***, *p* < 0.001, student’s test) triple asterisks (***) indicate significant difference in different treatments determined at *p* < 0.001 (student’s test)

The phytotoxic effects of EOs from *T. occidentalis* and the major compound (−)-α-thujone on the seed germination of *Arabidopsis thaliana* and *Taraxacum mongolicum* were investigated. As shown in Fig. 2, EOs from the *T. occidentalis* population at Shenyang (SY-EOs) exhibited higher inhibitory activity against the seed germination of *A. thaliana* than on that of *T. mongolicum*, with EC_50_ values of 3.81 ± 0.55 μg/mL and 61.86 ± 14.22 μg/mL, respectively. Notably, the pure compound (−)-α-thujone showed a higher inhibitory activity than did the EOs against the seed germination of *A. thaliana*, with an EC_50_ value of 1.22 ± 0.07 μg/mL. Moreover, (−)-α-thujone also obviously inhibited the seed germination of *T. mongolicum*, with an EC_50_ value of 73.91 ± 31.13 μg/mL, although this effect was weaker than inhibition of (−)-α-thujone of *A. thaliana* germination (Fig. 2).

### Seedling growth bioassay of EOs and (−)-α-thujone

**Fig. 3:**
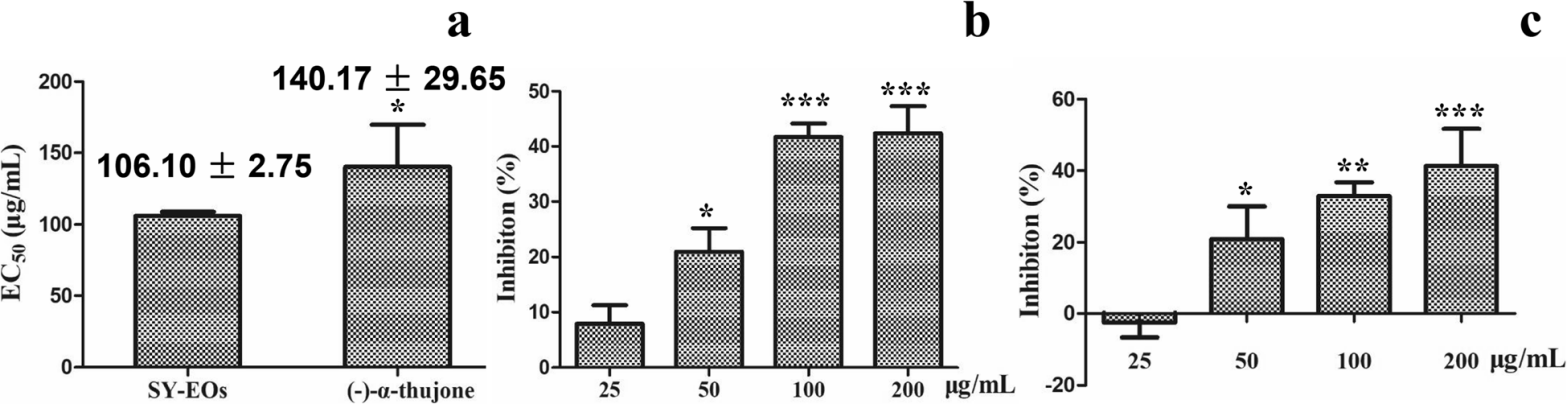
Effects of SY-EOs and (−)-α-thujone on the inhibition of *Taraxacum mongolicum* seedling growth (*n* = 4). **a** Effect of SY-EOs and (−)-α-thujone on inhibition of root elongation, (**b**) Effect of SY-EOs on weight of whole plant, (c) Effect of (−)-α-thujone on weight of whole plant. Single, double or triple asterisks (*, ** or ***) indicate significant differences in different treatments or between the 25 μg/mL and other treatments determined at *p* < 0.05, *p* < 0.01 or *p* < 0.001 (student’s test), respectively

EOs from *T. occidentalis* of the Shenyang population and pure (−)-α-thujone were also tested for their phytotoxic effect on the growth of *T. mongolicum* seedlings. Both EOs and (−)-α-thujone significantly inhibited root elongation in *T. mongolicum* seedlings, with EC_50_ values of 106.10 ± 2.75 and 140.17 ± 29.65 μg/mL, respectively (Fig. 3). Inhibition by SY-EOs was a dose-dependent up to a concentration of 100 μg/mL, and the maximum concentration of 200 μg/mL, a 40–50% reduction in the weight of whole plant was observed. The chemical, (−)-α-thujone showed similar inhibitor activity. However, at a concentration of 25 μg/mL, (−)-α-thujone seemed to slightly promote growth of *T. mongolicum* seedlings (Fig. 3)*.*

### Antifungal activities of EOs from Shenyang population

The EOs were also tested to determine whether they had any antifungal activity against four fungal strains. The fungal strains tested were the maize pathogens *Fusarium graminearum*, *Curvularia lunata*, and *Bipolaris maydis*, together with one human fungal pathogen *Candida albicans*. The *T. occidentalis* EOs displayed potent inhibitory activity against all four of these fungi, but were less potent than the positive control, Nystatin (Table 2).

**Table 2:** Antifungal activities of *T. occidentalis* EOs against pathogenic fungi

Strain of fungus	EOs of *T. occidentalis*	Nystatin as positive control
	IC_50_ μg/mL	IC_50_ μg/mL
*Fusarium graminearum*	284.96 ± 25.85	16.23 ± 3.10
*Curvularia lunata*	171.58 ± 91.42	1.02 ± 0.61
*Bipolaris maydis*	270.26 ± 53.49	5.56 ± 0.07
*Candida albicans*	286.76 ± 67.32	–

### Quantification (−)-α-thujone in the leaves

**Fig. 4:**
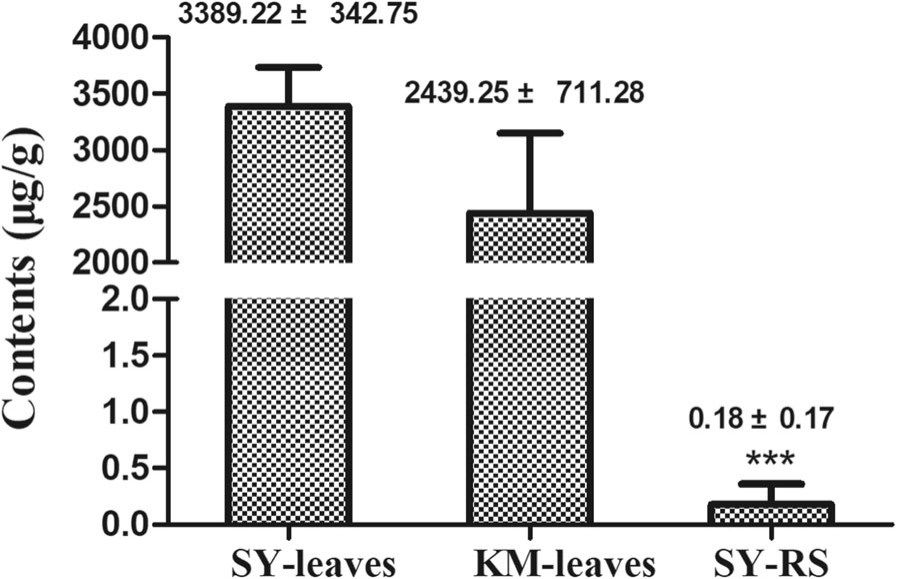
The content of (−)-α-thujone in leaves of *T. occidentalis* and rhizosphere soil*.* SY-leaf showed (−)-α-thujone in leaves of *T. occidentalis* in Shenyang (*n* = 5). KM-leaf showed (−)-α-thujone in leaves of *T. occidentalis* in Kunming (*n* = 3). SY-RS showed the (−)-α-thujone was extracted from the rhizosphere soil (RS) of plant distributed in Shenyang population (*n* = 5). Triple asterisks (***) indicate significant difference between SY-RS and SY-leaves determined at *p* < 0.001 (student’s test)

Since (−)-α-thujone was the major compound in the *T. occidentalis* EOs, we carried out a quantification to determine the concentration at which it is present in *T. occidentalis* leaves. Quantitative analyses were performed using GC-MS according to the external standard method. The concentration of (−)-α-thujone was found to be 3.39 ± 0.34 mg/g in the Shengyang population, with a similar value of 2.44 ± 0.71 mg/g in Kunming population (Fig. 4).

### Detection of compounds from EOs in rhizosphere soil

To determine whether compounds from EOs accumulated in the soil surrounding *T. occidentalis* in Shenyang, we collect rhizosphere soil at distances of 0.5, 1, 3, and 5 m from *T. occidentalis* plants. The soil was then analyzed as described above. The results show that (−)-α-thujone can only be detected in rhizosphere soil within 0.5 m of *T. occidentalis*, at a concentration of 0.18 ± 0.17 μg/g (Fig. 4).

## Discussion

It has been estimated that nearly 14% of the higher plant species across the world have been cultivated [31]. With the intervention of humans, the cultivated plants have been able to adapt to the new environments, including changing climatic conditions, diseases, and pests. *T. occidentalis*, grown as an ornamental plant, has shown good adaptability to many habitats in China, including subtropical Kunming and mid-temperate Shenyang. Using GC-MS analyses to obtain the chemical profiles of EOs from populations of this plant in Kunming and Shenyang, fourteen similar terpenoids have been identified, with the major constituents of the EOs in both populations being (−)-α-thujone, (+)-β-thujone, fenchone, rimuene, and (+)-hibaene. This suggests that climatic conditions do not have an effect on the main components in the EOs of *T. occidentalis*. Our results are consistent with those from other researchers studying *Thuja* across its range, who list monoterpenoids, sesquiterpenoids, and diterpenoids, especially (−)-α-thujone as the main constitutes in the *T. occidentalis* EOs [23, 32–34]. From our GC-MS and quantitative analyses, we found that (−)-α-thujone was the most abundant compound, with concentration of up to 3.39 ± 0.34 mg/g in the Shenyang population, and to 2.44 ± 0.71 mg/g in Kunming population (Fig. 4). Because the main components and contents of the EOs of *T. occidentalis* in Shenyang and Kunming were similar, EOs from the fresh leaves of *T. occidentalis* in Shenyang was further used for isolation and biological activity. (−)-α-thujone was isolated and identified by classical phytochemical methods to obtain 1 g of pure compound. In addition, a second major component, the diterpenoid (+)-hibaene was also isolated, and identified using NMR, and represents the first time this chemical has been reported from the EOs of *T. occidentalis* [35].

*T. occidentalis* has extensive and fascinating biological activity. Previous investigations have revealed that this plant has been widely used as a folk medicine to treat bronchial catarrh, cystitis, enuresis, psoriasis, and used both in homeopathy and evidence-based phytotherapy, suggesting that this plant has broad pharmacological potential [23]. Moreover, the known insecticidal effects of *T. occidentalis* EOs suggests that planting *T. occidentalis* might help against non-native insect in places where they are introduced [32–34]. These biological activities are likely to be related to the natural functions of EOs in plants in response to diverse biotic stresses. Exactly as we expected, the *T. occidentalis* EOs showed moderate antifungal activity, suggesting that the EOs might provide certain protection from infection by pathogenic fungi. Thujone can be produced by many trees and shows obvious toxicity against a number of organisms [36]. The inhibitory activity of (−)-α-thujone against the germination of *A. thaliana* seeds (EC_50_ value, 1.22 ± 0.07 μg/mL) was stronger than that against *T. mongolicum* germination (EC_50_ value, 73.91 ± 31.13 μg/mL), indicating model plant species *A. thaliana* is more sensitive than wild plants to this chemical. There were no significant differences between (−)-α-thujone and the EOs against the seed germination of *T. mongolicum*. Moreover, (−)-α-thujone exhibited a higher phytotoxic activity than that did the EOs against the seed germination of *A. thaliana*, indicating that (−)-α-thujone is an active constituent in the EOs of *T. occidentalis*. However, the higher inhibitory activity of the EOs than (−)-α-thujone against *T. mongolicum* seedling growth suggests the existence of other phytotoxic terpenoids in the EOs.

Interestingly, (−)-α-thujone, the most abundant and also potent constituent in *T. occidentalis* EOs, was also detected in rhizosphere soil underneath *T. occidentalis* trees, and has also been detected in the soil under western red cedar, *T. plicata* [37]. This suggests that this compound may be released into the environment by *T. occidentalis* as an allelochemical against other neighboring competitive plant species.

## Conclusions

Fourteen terpenoids have been identified as constituents of EOs from *T. occidentalis* growing in Kunming and Shenyang. Major constituents include thujone, fenchone, rimuene and (+)-hibaene, and in particular (−)-α-thujone. EOs displayed potent antifungal activity against four fungal strains. Significant phytotoxic effects of EOs and (−)-α-thujone against *A. thaliana* and *T. mongolicum* seed germination and seedling growth were also observed. In addition, the most abundant component of the EOs, (−)-α-thujone, was detected in rhizosphere soil. The results of this paper not only reveal the chemicals present in volatile EOs from *T. occidentalis* growing in different climatic conditions, but also provides more evidence of the phytotoxic activity of the EOs and (−)-α-thujone against seed germination and seedling growth, as well as a possible role of (−)-α-thujone as allelochemical for *T. occidentalis*.

## Methods

### Plant materials

The *Thuja occidentalis* plant material was collected from two locations: Kunming (E: 102° 74′, N: 25° 14′, three plants) and Shenyang (E: 123° 57′, N: 41° 82′, ten plants) in China in April 2018. Healthy trees about 5-years-old were selected to collect VOCs and extract EOs. Plants were provided and identified by Professor Wenshan Cui and the voucher specimens (SYNUB013056–SYNUB013060) were deposited at College of Bioscience and Biotechnology, Shenyang Agricultural University. Further plant material was collected from the Northern White-cedar Breeding Center of Tieling Development Area, as cut *T. occidentalis* branches were freely available following spring pruning. Wild-type of *Arabidopsis thaliana* (Col-0) was used for phytotoxic assay. Seeds from *Taraxacum mongolicum* Hand.-Mazz. were collected from close to a patch of northern white-cedar in the Northern White-cedar Breeding Center of Tieling Development Area, with the permission of Professor Wenshan Cui. *T. mongolicum* was identified by Professor Bo Qu and *T. mongolicum* voucher specimens (SYNUB014001–SYNUB014004) have been deposited at the College of Bioscience and Biotechnology, Shenyang Agricultural University.

### Collection of VOCs released from *T. occidentalis*

The VOCs released from the cultivated plants in Shenyang were collected using a closed-loop stripping system as described previously [27]. Briefly, each plant was placed inside a glass chamber capped with a closed loop stripping apparatus. Air flow was maintained by a vacuum pump. The VOCs were collected for 1 h using 150 mg Super Q traps (Supelco, PoraPak, Bellefonte, PA, USA). Each collective column was then eluted with 500 μL hexane into a gas chromatography sample vial. Each treatment was analyzed with five independent biological replicates.

### Extraction of EOs from leaves of *T. occidentalis*

The EOs were obtained by hydro-distillation with a Clevenger-type apparatus [38] using 25 g of fresh leaves from trees collected from both Kunming and Shenyang populations. The extraction was run for 1 h, and the oil obtained was subsequently dried over anhydrous sodium sulfate and refrigerated in a dark vial until it was analyzed and tested. Three replicates were run for Kunming population, while five replicates were carried out for Shenyang population.

### GC-MS analyses

Qualitative and quantitative analyses of EOs, VOCs, and extracts from rhizosphere soil were performed on a Shimadzu gas chromatograph-mass spectrometer (GC-MS) QP-2020 (Shimadzu, Japan) equipped with an autoinjector AOC-20is. The VOCs were separated using a Rtx-5MS column (30 mm × 0.25 mm, film thickness 0.25 μm) with helium as the carrier gas at a flow rate of 1.78 mL/min. 1 μL of each sample was injected in split mode (split ratio of 10:1) and the injector temperature was 250 °C. The oven temperature program was as follows: initial oven temperature of 40 °C, increased at 5 °C/min to 80 °C, after 4 min of hold time at 80 °C, followed by an increase of 10 °C/min to 280 °C and a hold time of 3 min. The mass detector, ion source, transfer-line and quadrupole temperatures were set at 230 °C, 250 °C, and 150 °C, respectively, with electronic impact (EI) mode at 70 eV and a scan range of *m/z* 50–500. A C_8_–C_40_ alkane standard solution (Sigma-Aldrich, USA) was analyzed for calculating retention indices (RIs) and monitoring system performance. Compounds were identified through comparison of their mass spectra with the reference spectra in NIST14 (Agilent Technologies, Palo Alto, CA, USA) and Wiley7n.1 MS-libraries (Wiley Publishing, Hoboken, NJ), as well as comparison of their RIs with published data (Kováts retention indices on unpolar column reported in literature (www.nist.gov). Target compounds were quantified by integration of peak areas and calibration.

### Isolation of main constituents of the EOs from the Shenyang population

About 50 mL EOs was collected by hydro-distillation using 6 kg fresh *T. occidentalis* leaves from the Shenyang population. These EOs were subjected to chromatography on silica gel columns, eluted with petroleum ether and petroleum ether: ethyl acetate (V/V, 100:1) to yield compounds **4** (about 1 g) and **14** (20 mg). The purity of **4** was more than 80% by GC-MS.

Spectra data of (−)-α-thujone (**4**): colorless oils, ^1^H NMR (Acetone-*d*_6_, 600 MHz) *δ*: 0.94 (3H, d, *J* = 6.8 Hz, H-8), 1.01 (3H, d, *J* = 6.8 Hz, H-9), 1.09 (3H, d, *J* = 7.5 Hz, H-10). ^13^C NMR (Acetone-*d*_6_, 150 MHz) *δ*: 30.3 (C-1), 39.8 (C-2), 219.6 (C-3), 47.8 (C-4), 26.2 (C-5), 19.1 (C-6), 33.7 (C-7), 20.2 (C-8), 19.9 (C-9), 18.2 (C-10).

Spectra data of (+)-hibaene (**14**), colorless oils, ^13^C NMR (Acetone-*d*_6_, 150 MHz) *δ*: 39.9 (C-1), 19.2 (C-2), 42.8 (C-3), 33.7 (C-4), 56.8 (C-5), 20.7 (C-6), 38.1 (C-7), 49.8 (C-8), 53.6 (C-9), 38.0 (C-10), 20.9 (C-11), 33.8 (C-12), 44.2 (C-13), 62.0 (C-14), 136.0 (C-15), 136.8 (C-16), 25.2 (C-17), 34.0 (C-18), 22.3 (C-10), 15.5 (C-20).

### Seed germination bioassay

Sterilized seeds of *Arabidopsis thaliana* and *T. mongolicum* were stored in a refrigerator at 4 °C for 2 days before use. The seeds were sown on MS medium [1.0% agar (w/v), pH 6.0] with the test samples of SY-EOs and (−)-α-thujone. The samples were tested for activity against *A. thaliana* at concentrations of 25, 20, 15, 10, 5, 1, and 0 μg/mL. Moreover, the results showed that there was no significant activity against *T. mongolicum* in the pretreatment of 25 μg/mL concentration. Thus, the samples were tested for activity against *T. mongolicum* at concentrations of 200, 100, 50, 25, and 0 μg/mL. These samples were first dissolved in DMSO, in order to have a final concentration of solvents not exceeding 0.5%. For each test, 15 seeds were distributed across moist MS medium in a Petri dish (6.0 cm diameter). Four duplicates of each concentration were carried out for each species. Seeds were allowed to germinate under a regime of 16 h light and 8 h dark at 22 °C (day) and 18 °C (night). The number of germinated seeds was checked daily until most seeds (≥90%) in the control Petri dishes had germinated. Germination counts were calculated over a period of 4 days. The EC_50_ was determined for the inhibition of seed germination.

### Seedling growth bioassay

*T. mongolicum* seeds were pretreated as described above. According to the results of seed germination, the samples were tested for activity against *T. mongolicum* at concentrations of 200, 100, 50, 25, and 0 μg/mL, and four replicates were carried out at each concentration. In each test, 15 seeds were placed in a horizontal line in a Petri dish (15 cm diameter). The Petri dishes were placed vertically in a growth chamber under a regime of 16 h of light and 8 h at 22 °C (day) and 18 °C (night). The length of the seedling roots and weights of whole seedlings were measured at 7 days after germination. The EC_50_ was determined for the inhibition of seedling roots.

### Antifungal assay

An antifungal activity assay was performed according to the broth microdilution method as described in the literature [39], with minor modifications. The EOs were tested for their in vitro antifungal activity against the fungal strains *Fusarium graminearum* (ACCC37120), *Curvularia lunata* (ACCC 38967), and *Bipolaris maydis* (ACCC 38948), and *Candida albicans*, all of which were obtained from the Agricultural Culture Collection of China. Two-fold serial dilutions of each sample, ranging from 256 to 4 μg/mL, were prepared in DMSO with a maximum concentration of 1% (v/v). Fungal strains were grown on potato dextrose agar at 28 °C for 1 week for the growth of spores. Potato dextrose broth was standardized to 10^5^ spores/mL. 100 μL of each spore suspension was then added into 96-well plates. The negative control plates were incubated with an equal volume of DMSO. Nystatin was used as positive control for antifungal activity. Five replicates were run for each treatment. After 48 h in an incubation shaker at 40 rpm, the wells of microtiter plate were read with a spectrophotometer at OD_625_ nm, and 50% inhibitory concentrations (IC_50_) were calculated.

### Detection of EO compounds in the rhizosphere soil around *T. occidentalis*

Rhizosphere soil was collected at distances of 0.5, 1, 3, and 5 m form the *T. occidentalis* plants and between 0 and 2 cm depth form the Shenyang population. After removing plant residues, the sample (5 g soil) was subjected to petroleum ether extraction with the addition of 30 mL petroleum ether and incubation in an ultrasonic bath for 30 min at room temperature, followed by centrifugation for 10 min at 12000 rpm. The supernatant was concentrated in vacuo. After the solvent had evaporated, the extraction was dissolved in 1 mL hexane and then analyzed using the same GC-MS method described above. Each treatment had five independent biological replicates.

### Quantification of (−)-α-thujone in the leaves and in the rhizosphere soil around *T. occidentalis*

Quantification of (−)-α-thujone in the leaves and rhizosphere soil around *T. occidentalis* was carried out using the same GC-MS method as described above, with the isolated authentic sample as the external standard. The leaf samples were taken from both the Shenyang and Kunming populations and were prepared as described above, while samples of rhizosphere soil were collected only from the Shenyang population. For quantification of (−)-α-thujone in the samples, a calibration curve for (−)-α-thujone was prepared. Triplicate injections were carried out at five concentrations (10, 5, 1, 0.5, and 0.1 μg/mL), and linear calibration curves were obtained by plotting the peak area versus concentration. Finally, the equation and correlation coefficient obtained from the linearity study for (−)-α-thujone (**4**) was *y* = 3E-06*x* - 0.2923 (R^2^ = 0.9997).

### Statistical analysis

The data are expressed as means ± SD of biological replicates. The statistical analysis of experimental data was conducted using Student’s *t* test run in SPSS 19.0. Values where *p* < 0.05 were considered significant.

## Data Availability

All data generated or analyzed during the current study are included in this published article.

## Abbreviations

EOs: Essential oils
DMSO: Dimethyl sulfoxide
GC-MS: Gas chromatography-mass spectrometer
IC: Inhibitory concentrations
KM: Plant population distributed in Kunming
NMR: Nuclear magnetic resonance
SY: Plant population distributed in Shenyang
SY-EOs: Essential oils of plant distributed in Shenyang population
SY-RS: Rhizosphere soil of plant distributed in Shenyang population
VOCs: Volatile organic compounds

## References

[1] Maffei ME, Gertsch J, Appendino G (2011). Plant volatiles: production, function and pharmacology. Nat Prod Rep.

[2] Kigathi RN, Weisser WW, Reichelt M, Gershenzon J, Unsicker SB (2019). Plant volatile emission depends on the species composition of the neighboring plant community. BMC Plant Biol.

[3] Pichersky E, Gershenzon J (2002). The formation and function of plant volatiles: perfumes for pollinator attraction and defense. Curr Opin Plant Biol.

[4] Pichersky E, Sharkey TD, Gershenzon J (2006). Plant volatiles: a lack of function or a lack of knowledge?. Trends Plant Sci.

[5] Sokame BM, Ntiri ES, Ahuya P, Torto B, Le Ru BP, Kilalo DC (2019). Caterpillar-induced plant volatiles attract conspecific and heterospecific adults for oviposition within a community of lepidopteran stemborers on maize plant. Chemoecology..

[6] Yip EC, Tooker JF, Mescher MC, De Moraes CM (2019). Costs of plant defense priming: exposure to volatile cues from a specialist herbivore increases short-term growth but reduces rhizome production in tall goldenrod (*Solidago altissima*). BMC Plant Biol.

[7] Penuelas J, Liusia J (2004). Plant VOC emissions: making use of the unavoidable. Trends Ecol Evol.

[8] Barney JN, Sparks JP, Greenberg J, Whitlow TH, Guenther A (2009). Biogenic volatile organic compounds from an invasive species: impacts on plant–plant interactions. Plant Ecol.

[9] Rahmani R, Andersson F, Andersson MN, Yuvaraj JK, Anderbrant O, Hedenström E (2019). Identification of sesquisabinene B in carrot (*Daucus carota* L.) leaves as a compound electrophysiologically active to the carrot psyllid (*Trioza apicalis* Förster). Chemoecology..

[10] Fan H, Li K, Yao F, Sun LW, Liu YJ (2019). Comparative transcriptome analyses on terpenoids metabolism in field- and mountain-cultivated ginseng roots. BMC Plant Biol.

[11] Padhy PK, Varshney CK (2005). Isoprene emission from tropical tree species. Environ Pollut.

[12] Courtois EA, Baraloto C, Paine CE, Petronelli P, Blandinieres PA, Stien D (2012). Differences in volatile terpene composition between the bark and leaves of tropical tree species. Phytochemistry..

[13] Zhang XH, Niu MY, Teixeira da Silva JA, Zhang YY, Yuan YF, Jia YX (2019). Identification and functional characterization of three new terpene synthase genes involved in chemical defense and abiotic stresses in *Santalum album*. BMC Plant Biol.

[14] Imbiscuso G, Trotta A, Maffei M, Bossi S (2009). Herbivory induces a ROS burst and the release of volatile organic compounds in the fern *Pteris vittata* L. J Plant Interact.

[15] Sergio R, Turlings TCJ (2010). Simultaneous feeding by aboveground and belowground herbivores attenuates plant-mediated attraction of their respective natural enemies. Ecol Lett.

[16] Barney JN, Hay AG, Weston LA (2005). Isolation and characterization of allelopathic volatiles from mugwort (*Artemisia vulgaris*). J Chem Ecol.

[17] Romagni JG, Dayan AFE (2000). Allelopathic effects of volatile cineoles on two weedy plant species. J Chem Ecol.

[18] Allard RW (1999). History of plant population genetics. Annu Rev Genet.

[19] Zhou J, Pan X, Xu H, Wang Q, Cui L (2017). Invasive *Eichhornia crassipes* affects the capacity of submerged macrophytes to utilize nutrients. Sustainability..

[20] Yu XJ, Ma KP (2010). Variation in reproductive characteristics of *Eupatorium adenophorum* populations in different habitats. Weed Res.

[21] Zheng G, Jia Y, Zhao X, Zhang F, Luo S, Li S (2012). *o*-Coumaric acid from invasive *Eupatorium adenophorum* is a potent phytotoxin. Chemoecology..

[22] Chang L, Song L, Park E, Luyengi L, Lee K, Farnsworth N (2000). Bioactive constituents of *Thuja occidentalis*. J Nat Prod.

[23] Naser B, Bodinet C, Tegtmeier M, Lindequist U (2005). *Thuja occidentalis* (arbor vitae): a review of its pharmaceutical, pharmacological and clinical properties. Evid-based Compl Alt.

[24] Hofmeyer PV, Seymour RS, Kenefic LS (2010). Production ecology of *Thuja occidentalis*. Can J For Sci.

[25] Dimitroula T, Konstantia G, Loretta POO, Miroslawa KB, Caroline S, Ioanna C (2009). Chemosystematic value of the essential oil composition of *Thuja* species cultivated in Poland-antimicrobial activity. Molecules..

[26] Oster U, Spraul M, Rüdiger W (1990). Natural inhibitors of germination and growth, V. possible allelopathic effects of compounds from *Thuja occidentalis*. Z Naturforsch C Biosci.

[27] Tholl D, Boland W, Hansel A, Loreto F, Rose US, Schnitzler JP (2006). Practical approaches to plant volatile analysis. Plant J.

[28] Sirisoma SN, Karin HM, John CE (2001). α- and β-Thujones (herbal medicines and food additives): synthesis and analysis of hydroxy and dehydro metabolites. J Agric Food Chem.

[29] Martín SA, Rovirosa J, Becker R, Castillo M (1980). Diterpenoids from *Baccharis tola*. Phytochemistry..

[30] Gniłka R, Szumny A, Białońska A, Wawrzeńczyk C (2012). Lactones 39. Chemical and microbial synthesis of lactones from (−)-α- and (+)-β-thujone. Phytochem Lett.

[31] Khoshbakht K, Hammer K (2008). How many plant species are cultivated?. Genet Resour Crop Evol.

[32] Szołyga B, Gniłka R, Szczepanik M, Szumny A (2014). Chemical composition and insecticidal activity of *Thuja occidentalis* and *Tanacetum vulgare* essential oils against larvae of the lesser mealworm, *Alphitobius diaperinus*. Entomol Exp Appl.

[33] Pavela R (2005). Insecticidal activity of some essential oils against larvae of *Spodoptera littoralis*. Fitoterapia..

[34] Kéïta SM, Vincent C, Schmidt JP, Arnason JT (2001). Insecticidal effects of *Thuja occidentalis* (Cupressaceae) essential oil. Can J Plant Sci.

[35] Kamdem P, Hanover J, Gage D (1993). Contribution to the study of the essential oil of *Thuja occidentalis* L. J Essent Oil Res.

[36] Hold KM, Sirisoma NS, Ikeda T, Narahashi T, Casida JE (2000). α-Thujone (the active component of absinthe): γ-aminobutyric acid type a receptor modulation and metabolic detoxification. Proc Natl Acad Sci U S A.

[37] Strobel B, Jensen PH, Rasmussen LH, Hansen HCB (2005). Thujone in soil under *Thuja plicata*. Scand J Forest Res.

[38] Avetisyan A, Markosian A, Petrosyan M, Sahakyan N, Babayan A, Aloyan S (2017). Chemical composition and some biological activities of the essential oils from basil *Ocimum* different cultivars. BMC Complem Altern M.

[39] Altıntop MD, Özdemir A, Turan-Zitouni G, Ilgın S, Atlı Ö, Demirel R (2015). A novel series of thiazolyl-pyrazoline derivatives: synthesis and evaluation of antifungal activity, cytotoxicity and genotoxicity. Eur J Med Chem.

